# A serum miRNA profile of human longevity: findings from the Baltimore Longitudinal Study of Aging (BLSA)

**DOI:** 10.18632/aging.101106

**Published:** 2016-11-07

**Authors:** Thalyana Smith-Vikos, Zuyun Liu, Christine Parsons, Myriam Gorospe, Luigi Ferrucci, Thomas M. Gill, Frank J. Slack

**Affiliations:** ^1^ Department of Molecular, Cellular and Developmental Biology, Yale University, New Haven, CT 06520, USA; ^2^ Yale School of Medicine, Department of Internal Medicine, New Haven, CT 06510, USA; ^3^ Bowdoin College, Brunswick, ME 04011, USA; ^4^ Intramural Research Program, National Institute on Aging, Baltimore, MD 21224, USA; ^5^ Institute for RNA Medicine, Department of Pathology, Beth Israel Deaconess Medical Center, Harvard Medical School, Boston, MA 02215, USA; ^6^ Graduate School of Arts and Sciences, Columbia University, New York, NY 10027, USA

**Keywords:** miRNA, aging, long-lived, short-lived, biomarker, longitudinal study

## Abstract

In *C. elegans*, miRNAs are genetic biomarkers of aging. Similarly, multiple miRNAs are differentially expressed between younger and older persons, suggesting that miRNA-regulated biological mechanisms affecting aging are evolutionarily conserved. Previous human studies have not considered participants' lifespans, a key factor in identifying biomarkers of aging. Using PCR arrays, we measured miRNA levels from serum samples obtained longitudinally at ages 50, 55, and 60 from 16 non-Hispanic males who had documented lifespans from 58 to 92. Numerous miRNAs showed significant changes in expression levels. At age 50, 24 miRNAs were significantly upregulated, and 73 were significantly downregulated in the long-lived subgroup (76-92 years) as compared with the short-lived subgroup (58-75 years). In long-lived participants, the most upregulated was miR-373-5p, while the most downregulated was miR-15b-5p. Longitudinally, significant Pearson correlations were observed between lifespan and expression of nine miRNAs (p value<0.05). Six of these nine miRNAs (miR-211-5p, 374a-5p, 340-3p, 376c-3p, 5095, 1225-3p) were also significantly up- or downregulated when comparing long-lived and short-lived participants. Twenty-four validated targets of these miRNAs encoded aging-associated proteins, including PARP1, IGF1R, and IGF2R. We propose that the expression profiles of the six miRNAs (miR-211-5p, 374a-5p, 340-3p, 376c-3p, 5095, and 1225-3p) may be useful biomarkers of aging.

## INTRODUCTION

Biomarkers of aging are biological parameters that change in a predictable direction with aging in most individuals and, when assessed early in life, may predict subsequent longevity better than chronological age alone. Beyond their prognostic utility, the discovery of biomarkers of aging is attractive because they may shed light into the intrinsic mechanism of aging as a biological process [1]. Identifying biomarkers of aging may also provide insight into the biological mechanisms that accelerate or decelerate aging [2]. Such biomarkers may be useful clinically for identifying persons at risk of developing adverse health outcomes traditionally associated with accelerated aging and to track the effectiveness of interventions aimed at slowing down the rate of aging and preventing its consequences such as multi-morbidity and disability.

miRNAs have emerged as important regulators of biological mechanisms that are relevant for aging. miRNAs are short non-coding RNAs that regulate gene expression generally by triggering mRNA decay and/or translational repression [3]. With over 1800 human miRNAs reported [4], miRNAs influence a wide range of biological functions, such as stem cell self-renewal, cell proliferation, apoptosis and metabolism [3].

Profiles of miRNAs found in plasma and serum have been linked to numerous cancers [5–8], cognitive impairment [9], Alzheimer's disease [10, 11] and other neurodegenerative disorders [12], and other pathologies [13], indicating that miRNAs are a new class of biomarkers of human diseases present in blood [14]. Because of the close relationship between these diseases and longevity, miRNAs may also serve as biomarkers of human aging. Our prior work has shown that miRNAs can serve as genetic biomarkers of aging in the nematode C. elegans [15]. Because miRNAs and aging genetic pathways are conserved from nematodes to humans, an increasing number of human miRNA studies have been carried out over the past several years. These studies have shown differential abundance of multiple miRNAs in peripheral blood mononuclear cells (PBMCs) or serum/plasma when comparing younger and older adults [16–21]. Sredni et al. found that changes in global miRNA levels, but not in mRNA levels, are associated with healthy aging in young adult women [16]. Noren Hooten et al. identified nine miRNAs that were differentially expressed in 30 vs. 64-year-old participants [17]. A summary of previous studies that have profiled miRNAs in biological samples of aged participants is provided in Table 1.

**Table 1:** Related works on miRNAs and human aging samples, 2010-present

Study	Samples	Analysis method	Participants' ages when miRNA expression was assessed	Main findings *
Sredni et al.	Whole blood	Illumina 96-sample Universal Matrix Array (739 miRNAs)	13 22-25 year olds (mean, 23.6 years) and 9 36-39 year olds (mean, 37.2 years)	The changes in global microRNA expression are associated with normal aging; the most differentially expressed microRNAs included miR-155, 8a, 142, 340, 363, 195, and 24
Noren Hooten et al.	PBMCs	Multiplex qRT-PCR (over 800 miRNAs total)	2 30 year olds and 2 64 year olds (male); validation in 14 young (mean, 30.1 years) and 14 old (mean, 64.2 years) individuals	9 miRNAs downregulated (miR-24, 103, 107, 128, 130a, 155, 221, 496, 1538)
Gombar et al.	B cells	Deep sequencing (284 miRNAs); qRT-PCR (validation)	3 63 year olds and 3 centenarians (female); validation in 27 individuals aged 50 to 100 years	22 miRNAs upregulated, 2 downregulated; miR-363 downregulated (validation)
ElSharawy et al.	Whole blood	Microarray (863 miRNAs); qRT-PCR (7 miRNAs, validation)	55 46 year olds and 15 centenarians and nonagenarians; validation in 17 younger (mean, 36.9 years) and 15 long-lived (mean, 101.5 years) individuals	16 miRNAs upregulated, 64 downregulated; 3 miRNAs (miR-106a, 126, 30d) downregulated (validation)
Serna et al.	PBMCs	Microarray (1105 miRNAs)	20 centenarians, 16 octogenarians, 14 young individuals	6 miRNAs upregulated (centenarians vs. young)
Olivieri et al.	Plasma	ABI TaqMan miRNA PCR array (365 miRNAs); qRT-PCR (validation)	11 20, 80, and 100 year olds; validation (only miR-21) in 111 healthy adults aged 20-105 (profiling cohort) and in 34 patients (mean, 87 years) with cardiovascular disease and 15 healthy centenarian offspring (mean, 72 years)	46 miRNAs downregulated, 12 up- then downregulated, 5 upregulated in profiling cohort; one miRNA (miR-21) downregulated in validation cohort
Noren Hooten et al.	Serum	Deep sequencing and qRT-PCR	20 young (mean, 30.1 years) and 20 old (mean, 64.2 years) individuals	3 miRNAs downregulated (miR-151a-5p, miR-181a-5p and miR-1248)

PBMCs, peripheral blood mononuclear cells.

* The findings were made from the perspective of the long-lived participants, such as centenarians. There was no overlap in findings between all 7 studies.

These studies vary in the types of samples used, groups of participants, methods of profiling miRNA expression, and number of miRNAs profiled. Perhaps because of these differences, there is no overlap in the identified miRNAs that are up- or downregulated in the older vs. younger participants. More importantly, because these studies were based on case-control or cross-sectional designs, the lifespans of participants were not known and longitudinal blood samples were not analyzed.

To address these limitations, we used miRNA PCR arrays to measure miRNA levels in serum samples obtained longitudinally at ages 50, 55, and 60 from 16 participants of the Baltimore Longitudinal Study of Aging (BLSA) who had documented lifespans. We compared miRNA expression changes not only across (i.e., between older and younger participants) but also within participants (using the three samples taken at different ages from each individual). In accordance with recent research that found a strong association between circulating miRNAs and human aging [22], our study suggests that circulating miRNAs are biomarkers of longevity.

## RESULTS

### Basic characteristics of the study participants

As shown in Table 2, the 16 participants were all non-Hispanic males who were non-smokers. Their lifespans ranged from 58 to 92 years, and the years of death ranged from 1998 to 2008. The majority of participants died of heart disease or cancer. The range of lifespans followed a sigmoidal curve similar to that of the 406 BLSA participants from which the sample was derived (data not shown). The average lifespan was 75.5 years, compared with 75.6 years for U.S. males from the United Nations' 2005-2010 life tables [23]; 8 participants had above average lifespan (long-lived subgroup: 76-92 years) and 8 had below average lifespan (short-lived subgroup: 58-75 years).

**Table 2:** Information from Baltimore Longitudinal Study of Aging (BLSA) regarding 16 participants in pilot study

No.	Visit number *	Age when sample was taken	Ethnicity	Smoking status	DOB	DOD	Lifespan	Cause of death (disease)
S1	12	53.1	White, not Hispanic Origin	former	1/20/1926	9/29/2004	78.7	heart disease
S1	14	57.2						
S1	17	63.1						
S2	4	51.1	White, not Hispanic Origin	former	12/23/1912	12/11/2004	92.0	cancer
S2	6	54.6						
S2	9	61.4						
S3	8	54.6	White, not Hispanic Origin	former	10/31/1917	2/18/2004	86.3	kidney neoplasm
S3	10	58.3						
S3	13	64.0						
S4	11	50.4	White, not Hispanic Origin	former	1/13/1929	5/4/2004	75.3	heart disease
S4	13	54.3						
S4	16	60.3						
S5	8	50.9	White, not Hispanic Origin	former	5/8/1924	9/14/2007	83.4	circulatory system disease
S5	11	57.1						
S5	13	61.7						
S6	10	50.0	White, not Hispanic Origin	former	1/31/1931	1/13/2007	76.0	respiratory system disease
S6	12	54.1						
S6	15	60.3						
S7	7	51.0	White, not Hispanic Origin	never	2/27/1923	1/8/1998	74.9	coronary heart disease
S7	10	57.0						
S7	13	63.0						
S8	7	52.6	White, not Hispanic Origin	former	9/1/1920	1/22/2004	83.4	nervous system disease
S8	9	55.8						
S8	13	61.7						
S9	5	50.5	White, not Hispanic Origin	former	6/8/1922	12/22/2004	82.5	nervous system disease
S9	12	67.1						
S9	13	69.3						
S10	13	50.1	White, not Hispanic Origin	former	10/13/1943	5/12/2001	57.6	circulatory system disease
S10	14	52.9						
S10	15	55.0						
S11	3	54.0	White, not Hispanic Origin	former	12/8/1918	1/3/2000	81.1	neoplasm
S11	9	70.0						
S11	10	72.5						
S12	9	53.2	White, not Hispanic Origin	never	5/8/1932	8/5/2003	71.2	circulatory system disease
S12	11	57.4						
S12	13	61.7						
S13	5	51.0	White, not Hispanic Origin	former	12/28/1924	2/11/1999	74.1	cancer
S13	7	55.0						
S13	10	61.0						
S14	10	52.0	White, not Hispanic Origin	former	8/14/1933	1/10/1998	64.4	cancer
S14	12	56.0						
S14	14	60.0						
S15	3	52.2	White, not Hispanic Origin	former	9/10/1935	12/6/1999	64.2	circulatory system disease
S15	4	57.7						
S15	7	63.1						
S16	1	54.3	White, not Hispanic Origin	former	8/30/1934	10/31/2007	73.2	neoplasm
S16	3	58.9						
S16	5	63.9						

DOB, date of birth; DOD, date of death.

* Participants were followed for life with follow-up visits conducted at intervals of 1–4 years, depending on the participant's age, e.g., with visits approximately every 2 years for persons aged 60 or older. The 16 individuals (identified by number such as S1, S2, etc.) in the pilot study have at least three serum samples from around age 50, 55, and 60 available for analysis.

### Magnitude of regulation and Pearson correlations

**Figure 1:**
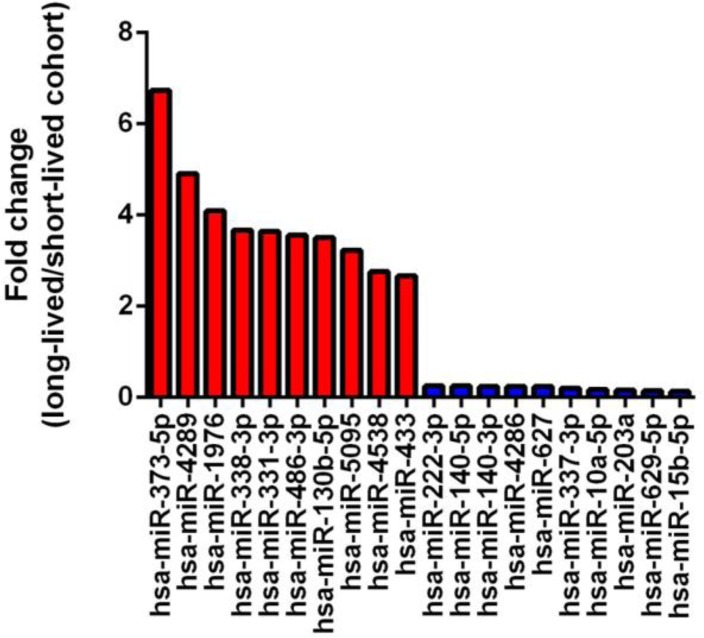
Fold change of 10 most upregulated and down-regulated miRNAs (long-lived vs. short-lived subgroup).

We compared the age 50 samples between the long-lived and short-lived subgroups by using methodology described in Figure S1 and S2. In total, we found 24 miRNAs that were significantly upregulated and 73 miRNAs that were significantly downregulated in long-lived participants. The 10 most upregulated and downregulated miRNAs are shown in Figure 1. The most upregulated miRNA was miR-373-5p, while the most downregulated miRNA was miR-15b-5p.

We focused our analysis on comparing the longer-lived to the shorter-lived subgroup (Figure S1), as this gave more significant results than comparing the longest-lived quartile and shortest-lived quartile to the “average lifespan” subgroup (Figure S3) or analyzing intra-individual miRNA expression (Figure S2). Pearson correlations between miRNA expression in participants' serum samples and participants' lifespan were calculated, and the results of miRNAs that overlapped between the three different control methods (spike-in, global average, or stably-expressed miRNAs) are shown in Table 3. A total of nine miRNAs had correlations with p-values<0.05. miR-5095 and miR-378g also had more than one significant correlation per dataset.

**Table 3:** Pearson correlations between miRNA expression in participants' serum samples and participants' lifespan *

miRNA	Spike-in Control			Global Average Control			Stable miRNAs Control		
	Dataset ^†^	R^2^	p-value	Dataset ^†^	R^2^	p-value	Dataset ^†^	R^2^	p-value
**hsa-miR-211-5p**	50	0.59	0.02	50	0.62	0.01	50	0.73	0.00
hsa-miR-29a-3p	55	0.27	0.04	55	0.25	0.05	(60-50)/50	0.44	0.01
**hsa-miR-374a-5p**	(60-50)/50	0.84	0.00	(60-50)/50	0.78	0.01	(60-50)/50	0.78	0.01
*hsa-miR-340-3p*^‡^	(55-50)/50	1.00	0.03	(55-50)/50	1.00	0.03	(55-50)/50	1.00	0.01
**hsa-miR-376c-3p**	55	0.91	0.04	55	0.94	0.03	60	0.65	0.02
**hsa-miR-5095**	60	0.31	0.05	60-50	0.46	0.02	60	0.39	0.03
							(60-50)/50	0.37	0.05
**hsa-miR-1225-3p**	60	0.28	0.03	60	0.29	0.03	60	0.27	0.04
hsa-miR-3622a-5p	55-50	0.37	0.03	55-50	0.41	0.03	60	0.29	0.04
				60-50	0.37	0.03	60-50	0.46	0.01
hsa-miR-378g	55-50	0.78	0.02	55-50	0.81	0.01	60	0.33	0.05
				50	0.35	0.04			

* Where p < 0.05 in all 3 control methods (p-values were rounded up to two decimal places).

‡ Italicized and underlined miRNA has perfect correlation, rounded up to two decimal places.

† Dataset refers to the type of analysis of samples taken at different ages, such as around age 50 or age 60. The change in expression level between samples taken at different ages was also examined. For example, (55-50)/50 indicates that the Ct value of the sample around age 55 was subtracted by that of the sample around age 50, and the difference was divided by the Ct value of the sample around age 50.

However, only six miRNAs (miR-211-5p, 374a-5p, 340-3p, 376c-3p, 5095, 1225-3p; in bold or italicized in Table 3) were correlated with lifespan and were significantly up or downregulated (above 2-fold or below 0.5-fold) when comparing subgroups of different lifespans, as shown in Figure S1 and S2. The correlations of these six miRNAs were reproducible in two separate experiments (data not shown). miR-340-3p had the highest correlation (all R^2^ values rounded up to two decimal places). miR-211-5p, miR-5095, and miR-1225-3p all had positive correlations and were similarly upregulated in the longer-lived subgroup but downregulated in the shorter lived subgroup. miR-374a-5p, miR-340-3p, and miR-376c-3p all had negative correlations and were similarly downregulated in the longer-lived subgroup but upregulated in the shorter lived subgroup. To illustrate these findings, Figure S4 shows an example of the correlation plot between the expression of miR-211-5p and lifespan for the age 50 subgroup; similar correlation plots were prepared for all six miRNAs.

### Bioinformatics

After confirming that the six miRNAs of interest were found on the miRandola circulating miRNA database [24], we explored the aging pathways that these miRNAs might target. By using both miRTarBase and miRWalk, we identified validated targets of the candidate biomarker miRNAs; only miR-5095 did not have any validated targets. Table 4 includes 24 aging-associated mRNAs that are validated targets of miR-211-5p, 374a-5p, 340-3p, 376c-3p, and 1225-3p when checking for overlap with GenAge. We found that miR-1225 had the most aging-associated targets at 25%, in which 3 out of 12 validated targets were also listed in the GenAge database [25]. About 14% (8/56) of the miR-374a validated target mRNAs also encoded aging-associated proteins, as well as ~8% (1/12) of the miR-376c targets, ~7% (3/41) of the miR-211 targets, and ~7% (10/146) of the miR-340 targets. miR-340 had the most validated targets and the most aging-associated targets overall. One target, *PARP1* mRNA [Poly(ADP-ribose) Polymerase 1], was found in the target lists of both miR-374a and miR-1225.

**Table 4:** Validated aging targets of five miRNAs from the pilot study

miRNA *	Validated aging target mRNAs ^‡^
miR-211	*CREB5*
	*DDIT4*
	*IGF2R*
miR-340	*LMNA*
	*ARHGAP*
	*MPHOSPH*
	*IFG2*
	*YWHAZ*
	*EEF1A1*
	*JUN*
	*PTEN*
	*CDKN2A*
	*HGF*
miR-374a	*EP300*
	*ATM*
	*HMGB2*
	*CISH*
	*PARP1*
	*BCL2*
	*TP73*
	*CDKN1A*
miR-376	*IGF1R*
miR-1225	*JUND*
	*PARP1*
	*PRDX1*

* miR-5095 was not listed here because it did not have any validated aging targets.

‡ miRTarBase + miRWalk, overlap with GenAge. Note that *PARP1* (Poly(ADP-ribose) Polymerase 1) mRNA is a validated target of both miR-374a and miR-1225.

## DISCUSSION

We conducted a pilot study of miRNAs as biomarkers of aging by analyzing miRNA expression in serum samples from a longitudinal human aging study. We found that the expression of six circulating miRNAs in mid-adulthood significantly correlates with subsequent longevity, suggesting that these miRNAs may be useful biomarkers of human aging. As far as we know, this is the first study that directly correlates miRNA with human longevity using data from a longitudinal study.

Many interesting expression profiles were observed between study participants with different lifespans. For example, when comparing samples analyzed at age 50 between the long-lived and short-lived subgroups, we identified the 10 most differentially higher and lower expressed miRNAs (Figure 1). The most upregulated miRNA in long-lived participants, miR-373-5p, is part of the miR-373 family, which functions as a tumor suppressor in breast cancer [26]. The most down-regulated miRNA in long-lived participants, miR-15b-5p, has been found to be upregulated in oral cancer cells [27]. Because lifespan is a complex trait characterized by escaping, delaying, or surviving fatal age-related diseases, including cancers, further scrutiny of the potential roles of the identified miRNAs in human aging is of great importance and interest.

The novel approach for the current study was to perform Pearson correlations between miRNA expression in all serum samples and the lifespan of 16 participants. Nine miRNAs had correlations with p values<0.05 (Table 3). Four of these miRNAs (miR-374, 376, 29, 378) have been shown in prior studies to be differentially expressed between older and younger persons in separate profiling experiments [18, 20, 21]. miR-374 was also downregulated in older participants in an earlier study [18], but in contrast with our study, miR-376 was upregulated in older participants in another report [20]. The trend in expression changes of the other two miRNAs (miR-29, 378) in prior studies was similar to our findings [20, 21].

Six of the nine miRNAs (miR-211-5p, 374a-5p, 340-3p, 376c-3p, 5095, 1225-3p) may serve as useful biomarkers, as each of the six miRNAs were correlated with lifespan and were significantly up- or down-regulated. Future studies can identify how examining expression of multiple miRNAs simultaneously versus one or a few miRNAs individually would affect these correlations. While some miRNA biomarker or disease-association studies have found significant correlations only by analyzing a profile of expression of multiple miRNAs [11, 28], our study did identify miRNAs that individually correlate with lifespan. Further, it is striking that miRNA expression at ages 50, 55, and 60 correlates with the eventual, quite varied lifespans of the 16 participants in our pilot study.

Most of the 16 participants died from either heart disease or cancer (Table 2), and 15 participants were relatively healthy at the time of blood draw and only had coronary artery disease. The current study was not designed to evaluate the association between miRNA expression and disease-specific mortality. Nonetheless, our results suggest that these miRNAs may have separate functions during the process of aging itself. Interestingly, none of the six miRNAs has previously been shown to play a mechanistic role in aging, and none has been implicated in heart disease, but many have been shown to function and/or act as biomarkers in different cancers (miR-211: melanoma cell invasive-ness; head, neck, renal cell carcinomas; pancreatic cancer; miR-374: small cell lung cancer; miR-340: osteosarcoma, colorectal cancer, breast cancer, gastric cancer; miR-376: glioblastoma, hepato-cellular carcino-ma) [24].

We identified 24 aging-associated mRNAs that are also validated targets of the five miRNAs (the sixth miRNA, miR-5095, did not have any validated targets). Notably, *PARP1* mRNA, found in target lists of both miR-374a and miR-1225, encodes PARP1, a protein known for its role in repairing single-strand breaks during DNA replication [29, 30]. PARP1 has also been linked to aging, being present in a complex with WRN DNA repair proteins that are deficient in participants with Werner syndrome, a premature aging syndrome [31]. Additionally, one study found that there was higher PARP activity in cell lines established from blood samples of centenarians compared with younger participants [32]. PARP activity (due to PARP1) measured in blood samples of 13 mammalian species is also associated with maximum lifespan [33], suggesting an evolutionary role of PARP1 in determining species-specific lifespan. These findings indicate that PARP1 repair activity regulates mammalian longevity, consistent with the DNA damage theory of aging [34]. Additionally, both *IGF1R* mRNA (a miR-376c target) and *IGF2R* mRNA (a miR-211 target) encode receptors of the insulin signaling pathway (IGF1R and IGF2R, respectively), which has been tightly linked to variation in human longevity[35, 36]. By examining aging-related roles of their genetic targets, we have identified a functional context for how these miRNAs might regulate aging processes. Additional research is needed to confirm correlations between the expression of these miRNAs and that of their target mRNAs, and to fully elucidate genetic regulation mechanisms within aging-related pathways.

**Figure 2:**
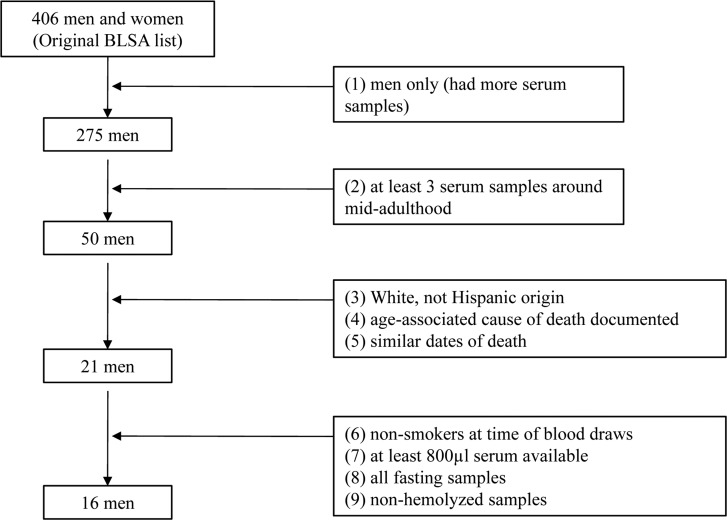
Flowchart showing the assembly of study participants for the pilot study.

Calculating Pearson correlations and separating participants into longer-lived vs. shorter-lived subgroups were only possible because we had obtained serum samples from a longitudinal study. This is a unique strength of the current study relative to prior miRNA profiling studies that have used blood samples from a single point in time among aged participants (Table 1). However, one limitation of our study is the relatively small sample size. Because this was a pilot study, we wanted to reduce the heterogeneity of our sample. This was accomplished through the use of several entry criteria (Figure 2), leading to the inclusion of 16 eligible men. Because of the small sample size, we did not account for multiple comparisons. Further validation of our results, especially in women and non-Whites, with larger sample sizes is needed.

Our study has implemented a novel approach to identify human aging biomarkers in an attempt to translate basic scientific discoveries in model organisms to human aging. Our results suggest that the expression profiles of six miRNAs may be useful biomarkers of aging. Although preliminary, these results provide a basis for investigating miRNAs as potential predictors of future longevity, and they highlight the potential roles of select miRNAs in regulating aging processes, thus warranting further validation and mechanistic explorations.

## MATERIALS AND METHODS

### Study design and setting

The Baltimore Longitudinal Study of Aging (BLSA) is a longitudinal study of human aging that began in 1958 with more than 1400 volunteers, ranging in age from 20s to 90s [37]. A detailed description of the BLSA has been provided previously [37, 38]. Briefly, participants were assessed at the NIA Clinical Research Unit in Baltimore, Maryland by certified nurse practitioners and technicians following standardized protocols. The assessments included physiological parameters, biomarkers, risk factors, disease-related measures, impairments, and physical and cognitive function. Participants were followed for life with follow-up visits conducted at intervals of 1–4 years, depending on the participant's age, with visits approximately every 2 years for older persons aged 60 to 80 years and visits every year thereafter. All participants provided signed informed consent, and the BLSA protocol is approved by the NIEHS Institutional Review Board.

### Participants

Longitudinal serum samples for this pilot study were obtained for 16 BLSA participants. The inclusion criteria were: male, availability of blood samples at around age 50 with at least three samples between ages 50 and 60, at least 800 μl of available serum, all fasting and non-hemolyzed samples (preventing introduction of miRNAs from red blood cells [39]), having a known cause of death, non-Hispanic White, and non-smoker (as miRNA dysregulation is linked to smoking-related diseases [40]) at the time of the blood draws. The assembly of participants for this study is provided in Figure 2. Eventually, we selected 16 participants (48 serum samples), all of whom had three blood samples taken within the range of ages 50, 55, and 60 (Figure S5). The sample was restricted to men because an inadequate number of women met the inclusion criteria.

### miRNA extraction and profiling

The Serum/Plasma miRNA isolation kit, miScript II RT Kit (using HiSpec buffer), and miScript SYBR Green PCR Kit (Qiagen) were used to prepare samples for profiling following standard protocols. The miScript Human Serum & Plasma 384HC miRNA PCR Arrays (Qiagen) were used to profile 372 miRNAs and 12 controls (6 snoRNA/snRNA controls, a miRNA reverse transcription control, a positive PCR control, and a *C*. *elegans* miR-39 spike-in) for each of the 48 serum samples. A Roche LightCycler 480 machine was used to perform qPCR. Using the miScript miRNA PCR Array Handbook, the handbook protocol determined the same baseline and threshold across all amplification plots, in which the earliest amplification was manually set to cycle 15 and the threshold was set to 0.1. To confirm qRT-PCR results from the first RNA isolation, RNA was isolated a second time from serum in separate tubes from the same participants. In this second experiment, miScript Primer Assays were ordered to separately confirm expression of six identified miRNAs of interest (please see Ct analysis and Results section), along with spike-in control (miR-39) and four stable miRNAs (miR-21, 122, 126, 574) by following standard protocols.

### Ct analysis

Data were analyzed using Sabio Sciences software to evaluate Ct values of controls before calculating fold changes. The miScript miRNA PCR Array Handbook advises that if the array is unbiased, normalize to the mean Ct of all expressed targets on the plate or to the mean Ct of at least four commonly expressed targets; however, we decided to perform both normalization methods, as well as normalizing to the spike-in control (see Data S1). The four miRNAs (miR-21, 122, 126, 574) with stable Ct values across all 48 serum samples were chosen from a list of stable miRNAs used for normalization by previous miRNA profiling studies using blood samples from aged participants [17–21, 41, 42]. The snoRNA and snRNA controls provided in each Qiagen miScript Human Serum & Plasma 384HC miRNA PCR Array were not used because the Ct values were all above 35 or were inconsistent on the same array. In the second experiment (referenced above), the mean Ct of all expressed targets was not used as a control due to using different assays (see supplementary materials).

The method for calculating fold regulation for a given miRNA (repeated for each miRNA) among different age groups is described in detail in Figure S1 and S3. For example, comparing the long-lived with short-lived subgroup identified up- and downregulated miRNAs between the two subgroups (above 2-fold or below 0.5-fold). As shown in Figure S2, Pearson correlations were calculated using raw Ct values normalized to the appropriate controls (the number of controls varying depending on the experiment), and correlations were calculated between the Ct values and lifespans of the 16 participants. Only Pearson correlations that were statistically significant (p<0.05) using all controls were analyzed. Pearson correlations for each miRNA Ct value and the participant's lifespan were calculated using Ct values from samples taken at different ages, including around age 50, 55, and 60. These calculations were repeated for 60-55 (the Ct value of the sample around age 60 was subtracted from that of the sample around age 55), 60-50, (55-50)/50 (the Ct value of the sample around age 55 was subtracted from that of the sample around age 50, and the difference was divided by the Ct value of the sample around age 50), and (60-50)/50. For each miRNA, any significant correlation from any of these Ct values was analyzed.

### Bioinformatics

By using both miRTarBase and miRWalk, we identified validated targets of the candidate biomarker miRNAs. We then checked for overlap with a collection of 300 genes (300 mRNAs) listed in GenAge: The Ageing Gene Database from Human Ageing Genomic Resources [25, 43] to determine if any of the mRNA targets of the remaining miRNAs might have aging-associated functions. The GenAge database includes a collection of genes associated with longevity and/or aging in model organisms (e.g., flies, mice) and humans.

## SUPPLEMENTARY MATERIAL FIGURES


