# Supplementary material for: A serum miRNA profile of human longevity: findings from the Baltimore Longitudinal Study of Aging (BLSA)
Source: Aging (Albany NY). 2016 Nov 7;8(11):2971–83. doi: 10.18632/aging.101106 (PMC5191881; doi:10.18632/aging.101106)
Supplement: Supplementary file 1 [file aging-08-2971-s001.pdf]

SUPPLEMENTARY MATERIAL

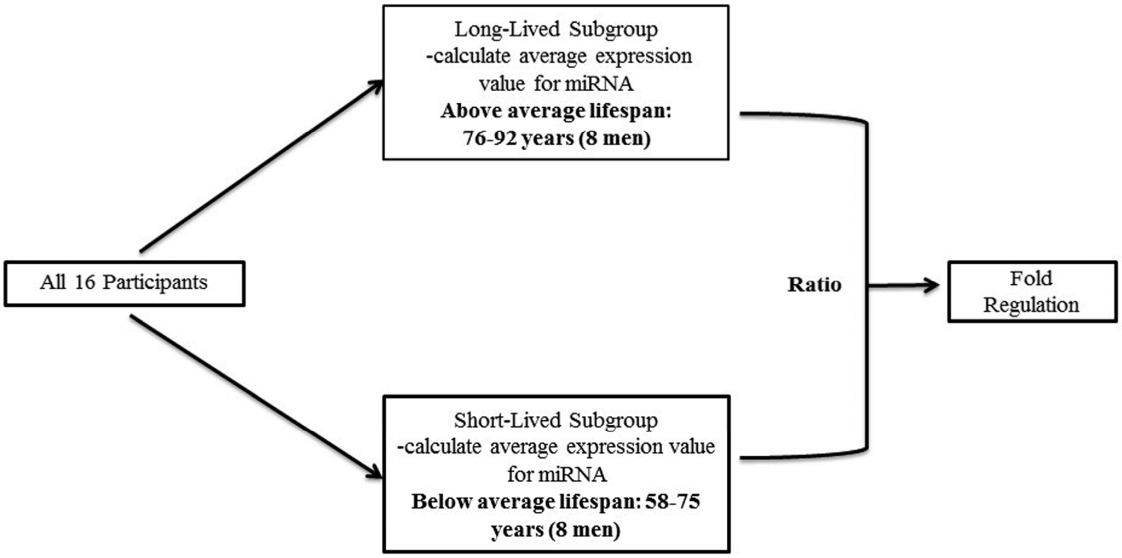

Figure S1. Method for calculating fold regulation for a given miRNA (repeated for each miRNA) by comparing long-lived subgroup to short-lived subgroup. A total of 304 miRNAs had Ct values below 35 across all 48 arrays.

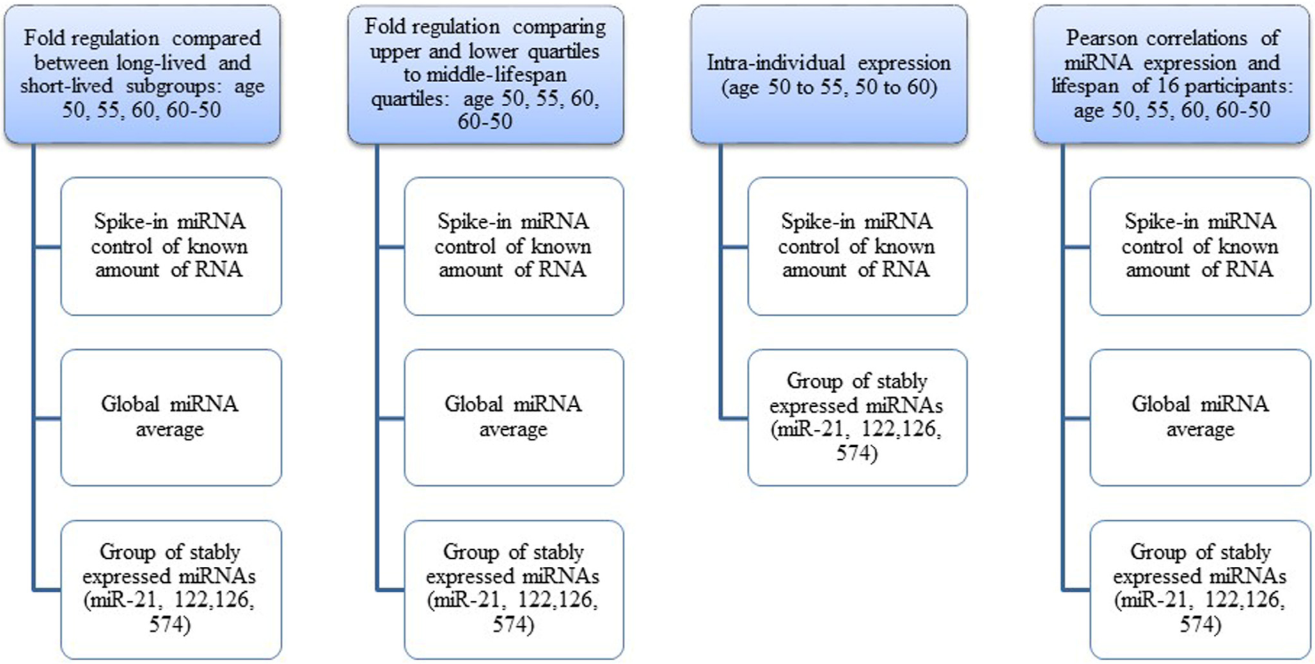

Figure S2. Flowchart of four different methods for analyzing serum samples.

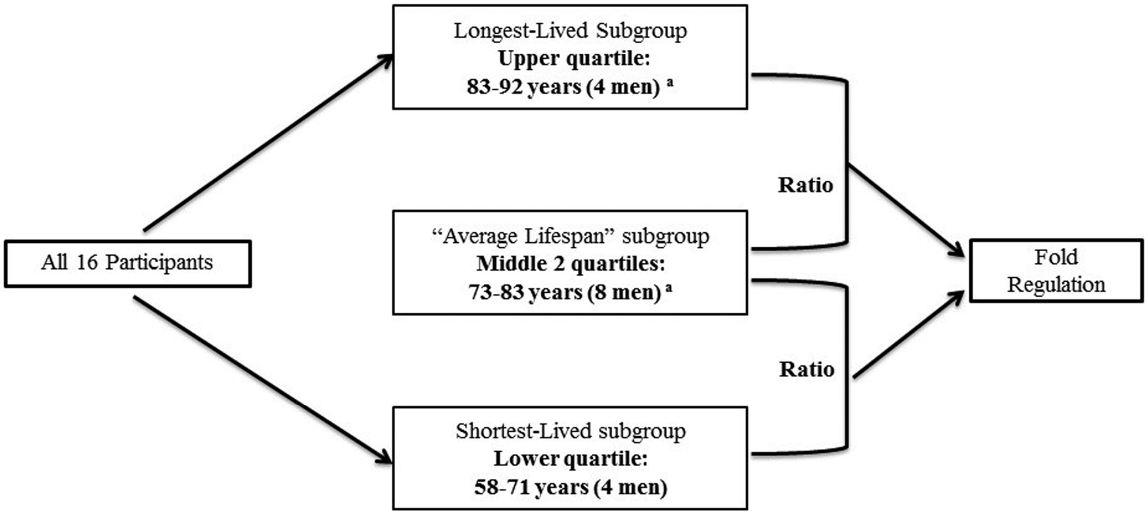

Figure S3. Method for calculating fold regulation for a given miRNA (repeated for each miRNA) by comparing longest-lived subgroup and shortest-lived subgroup to "average lifespan" subgroup.

[a] Sample S5 and sample S8 had lifespans of 83.4 and were categorized into the longest-lived subgroup. Sample S9 had a lifespan of 82.5 and was categorized into the average lifespan subgroup. A total of 304 miRNAs had Ct values below 35 across all 48 arrays.

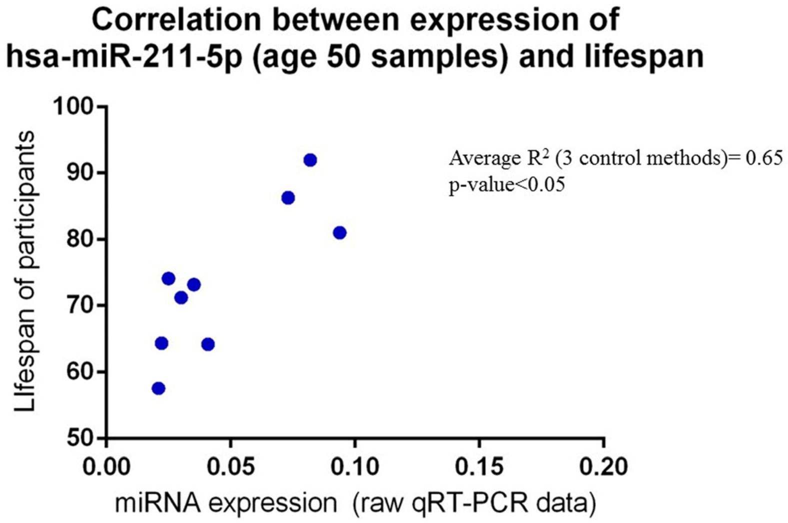

Figure S4. Correlation between the expression of miR-211-5p and lifespan (age 50 samples).
R^2 values from Table 3 were averaged. Ct values for some of the 16 participants were not detected or were above 35 and are thus not plotted. Dataset for Ct values obtained from Table 3.

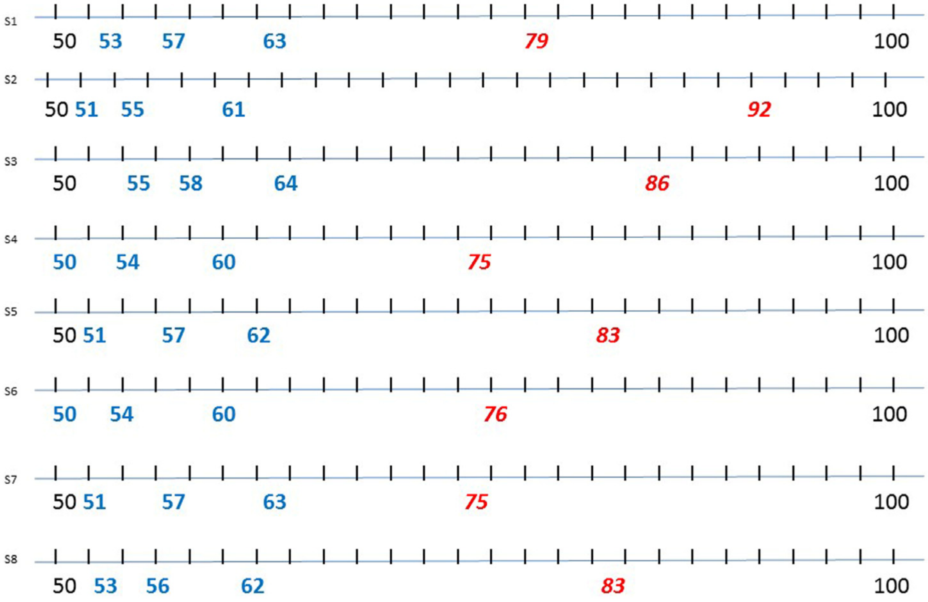

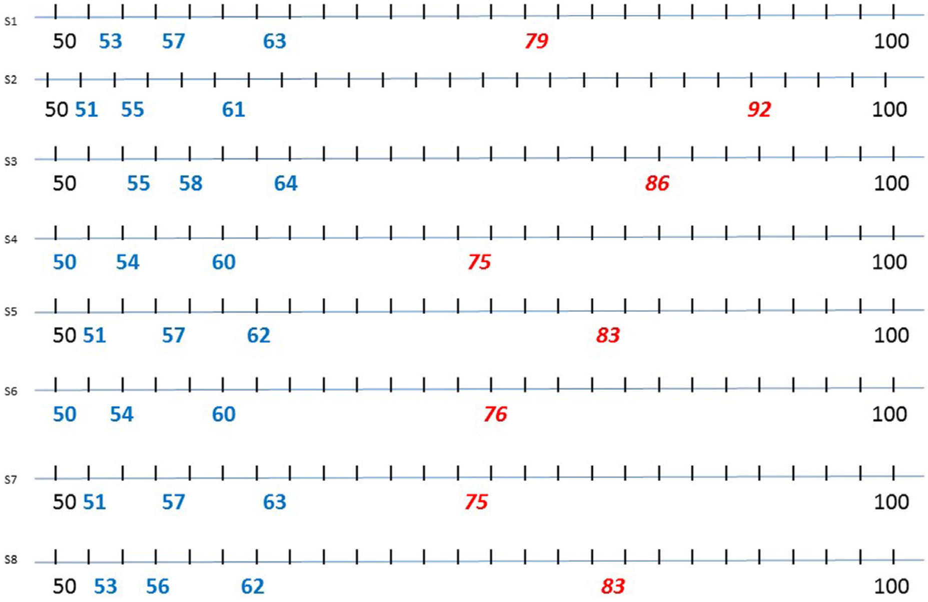

Figure S5. Timeline of serum samples from 16 BLSA participants. Red numbers indicate the lifespan of the participants. Blue numbers indicate the timepoints when the serum samples were taken. Participants are listed in order by number (e.g., S1, S2...). We profiled miRNA expression from three serum samples from each participant. The three samples were taken as close as possible to age 50, 55, and 60 for each participant, based on availability. Only two participants, S9 and S11, did not have available samples within the range of age 50, 55, and 60 because their corresponding serum samples at those ages were in short supply. One participant, S10, died before age 60; thus, we used serum samples slightly earlier than this time point. However, the samples that we did profile for these participants still exhibited similar expression patterns that followed the trends we observed with the other 13 participants.

Ct analysis

Raw Ct values were normalized using three separate Ct values: 1) average of all 304 miRNAs, 2) average of four stable miRNAs, and 3) C. elegans miR-39 spike-in. Thus, the miRNAs were normalized to the controls found on the same plate, not between plates. Using the first round of RNA isolated from serum, 304 miRNAs out of the 372 miRNAs on the custom arrays had consistent Ct values below 35 across all 48 plates; thus, these 304 miRNAs were used for further analysis. Using the second round of RNA isolated from serum, only the six miRNAs of interest (with significant Pearson correlations from the first qRT-PCR experiment) were analyzed using individual assays. The six miRNA assays were quantified only using the spike-in control in order to perform all qRT-PCR reactions on a single plate. Lastly, in the final qRT-PCR experiment, six plates (one for each miRNA assay) each contained the spike-in control and the four stable miRNAs to normalize within each plate.

The method for calculating fold regulation for a given miRNA (repeated for each miRNA) in order to provide a ratio of miRNA expression in long-lived subgroup compared with short-lived subgroup is shown in Fig. S1. Similarly, an alternative method for calculating fold regulation for a given miRNA (repeated for each miRNA), in order to provide a ratio of miRNA expression in long-lived subgroup and short-lived subgroup compared to the two "average lifespan" subgroups is shown in Fig. S3. Comparing the long-lived with short-lived subgroup, or the long-lived to middle-age subgroup and the short-lived to middle-age subgroup, identified up- and down-regulated miRNAs between subgroups (above 2-fold or below 0.5-fold).

Fig. S2 describes the flowchart of four different methods for analyzing serum samples taken at different ages (e.g., around age 50). The first two methods are described in Fig. S1 and Figure S3. The third method involves comparing miRNA expression from samples from the same individual, but there were no significant expression changes here. The fourth and final method involves correlating raw miRNA Ct values to individuals' lifespans. The raw Ct values were separately normalized to a spike-in control, global average, and group of stably expressed miRNAs; only miRNAs that had the same Ct values across all three normalization methods were analyzed. Notably, Pearson correlations for each miRNA Ct value and the individual's lifespan were performed using Ct values from samples taken at different ages, including around age 50 (e.g., age 51 was considered as age around 50), 55, 60, 60-55 (the Ct value of the sample around age 60 was subtracted by that of the sample around age 55), 60-50 (the Ct value of the sample around age 60 was subtracted by that of the sample around age 50), (55-50)/50 (the Ct value of the sample around age 55 was subtracted by that of the sample around age 50, and the difference was divided by the Ct value of the sample around age 50), and (60-50)/50 (the Ct value of the sample around age 60 was subtracted by that of the sample around age 50, and the difference was divided by the Ct value of the sample around age 50). Any significant correlation from any of these Ct values for each miRNA was analyzed.
